# Understanding How Commensal Obligate Anaerobic Bacteria Regulate Immune Functions in the Large Intestine

**DOI:** 10.3390/nu7010045

**Published:** 2014-12-24

**Authors:** Eva Maier, Rachel C. Anderson, Nicole C. Roy

**Affiliations:** 1 Food Nutrition & Health Team, Food & Bio-based Products Group, AgResearch Grasslands, Palmerston North 4442, New Zealand; E-Mails: eva.maier@agresearch.co.nz (E.M.); nicole.roy@agresearch.co.nz (N.C.R.); 2 Riddet Institute, Massey University, Palmerston North 4474, New Zealand; 3 Gravida: National Centre for Growth and Development, The University of Auckland, Auckland 1142, New Zealand

**Keywords:** intestinal microbiota, intestinal immune homeostasis, obligate anaerobic bacteria, *Faecalibacterium prausnitzii*, *Bacteroides thetaiotaomicron*, *Bacteroides fragilis*, *Akkermansia muciniphila*, segmented filamentous bacteria, dual-environment co-culture models

## Abstract

The human gastrointestinal tract is colonised by trillions of commensal bacteria, most of which are obligate anaerobes residing in the large intestine. Appropriate bacterial colonisation is generally known to be critical for human health. In particular, the development and function of the immune system depends on microbial colonisation, and a regulated cross-talk between commensal bacteria, intestinal epithelial cells and immune cells is required to maintain mucosal immune homeostasis. This homeostasis is disturbed in various inflammatory disorders, such as inflammatory bowel diseases. Several* in vitro* and* in vivo* studies indicate a role for *Faecalibacterium prausnitzii*, *Bacteroides thetaiotaomicron*, *Bacteroides fragilis*,* Akkermansia muciniphila* and segmented filamentous bacteria in maintaining intestinal immune homeostasis. These obligate anaerobes are abundant in the healthy intestine but reduced in several inflammatory diseases, suggesting an association with protective effects on human health. However, knowledge of the mechanisms underlying the effects of obligate anaerobic intestinal bacteria remains limited, in part due to the difficulty of co-culturing obligate anaerobes together with oxygen-requiring human epithelial cells. By using novel dual-environment co-culture models, it will be possible to investigate the effects of the unstudied majority of intestinal microorganisms on the human epithelia. This knowledge will provide opportunities for improving human health and reducing the risk of inflammatory diseases.

## 1. Introduction

The health of humans is impacted by the symbiotic relationship with their microbiota, while a dysbiosis of the resident microbiota is associated with various disorders, such as inflammatory bowel diseases [1,2,3], colorectal cancer [4,5], coeliac disease [6] and obesity [7]. The intestinal mucosa is in intimate contact with commensal microorganisms and food antigens but also faces the constant risk of infection by pathogens from the external environment. The challenging task for the intestinal immune system is to maintain a homeostatic balance between tolerance towards harmless agents and immunity against pathogens. This is achieved through various regulatory adaptations in the cross-talk between the commensal bacteria, intestinal epithelial cells (IECs) and immune cells of the GIT.

Even though the importance of the intestinal microbiota for human health is widely recognised, mechanistic studies explaining how commensal bacteria exert their effects remain limited. The majority of the research on host-microbiota interactions has focused on oxygen-tolerant bacteria. This is partially a consequence of the difficulty of culturing obligate anaerobic commensal bacteria that cannot survive in the presence of oxygen together with human epithelial cells that require oxygen for survival. However, over 99% of the bacterial species residing in the large intestine are obligate anaerobic bacteria [8], which means that the majority of the effects of the microbiota on the large intestine remain unknown.

In this review, the importance of the intestinal microbiota for the development and function of the immune system and its role in promoting immune homeostasis in the GIT are discussed. Particularly, the mechanisms used by five prominent commensal obligate anaerobes to modulate immune functions and maintain a healthy intestine are described. Finally, this review describes novel models that will enable the study of the interactions between the host and its mainly obligate anaerobic microbiota* in vitro*. This new knowledge will ultimately provide opportunities to improve both intestinal and overall health.

## 2. The Human Intestinal Microbiota

The human GIT is colonised by an estimated 10^14^ microorganisms, with microbial cells outnumbering human cells by a factor of ten [9]. Some consider the intestinal microbiota to be a human microbial “organ” [8]. This complex system consists of a diverse community, which is dominated by bacteria, but also contains archaea, eukaryotes (e.g., fungi, protozoa) and viruses [10]. Most bacterial species colonising the human GIT belong to the phyla *Firmicutes* and *Bacteroidetes*, while species of the phyla *Actinobacteria*, *Proteobacteria* and *Verrucomicrobia* exist in lower numbers [8]. The number of bacterial species within the human intestinal microbiota has often been estimated to be in the range of 500 to over 1000 species [8,11]. With an increasing number of metagenomic sequencing studies undertaken over recent years, the intestinal microbiota is often defined based on its genetic content. Results of the Metagenomics of the Human Intestinal Tract (MetaHIT) project revealed an approximate total of 3.3 million non-redundant microbial genes in the human microbiota derived from faecal samples of 124 European individuals; approximately 150 times more genes than the human genome [10]. From this result it was estimated that the intestinal microbiota of the entire cohort comprised 1000 to 1150 prevalent (more frequent) bacterial species, with at least 160 prevalent bacterial species per person. Furthermore, this study demonstrated the predominance of bacteria within the human microbiota as 99% of the genes were of bacterial origin. The rest were mostly archaeal and only 0.1% of the genes were of eukaryotic and viral origins [10].

Due to their anaerobic nature, the majority of the bacterial strains in the GIT are difficult to culture using standard laboratory techniques. Therefore, progress in molecular biology techniques including 16S rRNA gene and metagenomic sequencing has been crucial in unravelling the diversity of the intestinal microbiota [8,10].

The composition and density of bacteria changes along the GIT. Bacterial numbers increase along the GIT from 10^3^ to 10^5^ bacteria per mL of luminal contents in the stomach and duodenum to approximately 10^9^ to 10^12^ per mL in the ileum and colon, respectively [12,13]. Additionally, bacterial diversity increases from proximal to distal locations of the GIT. The stomach is often colonised by species of *Helicobacter* and to a lesser extent *Prevotella*, *Streptococcus*, *Veillonella*, and *Rothia*, which is a genus within the family *Actinomycetaceae* [14,15]. The genera *Streptococcus*, *Lactobacillus*, *Enterococcus* and members of the *Enterobacteriaceae* family are major constituents of the microbiota in the small intestine [16].

The microbiota composition in the colon is more diverse and shows considerable variation between individuals. However, several studies proposed that the microbial communities can be divided into distinct clusters. For example, three predominant variants were suggested to exist, which were designated as enterotypes [17]. These three enterotypes are either enriched in *Bacteroides* (enterotype 1), *Prevotella* (enterotype 2) or *Ruminococcus* (enterotype 3). Enterotypes were shown to be influenced by long-term diet [18]. A more recent study identified four distinct community types within bacteria from stool samples [19]. Community type A was characterised by the highest levels of *Bacteroides* but a lack of *Prevotella* and *Ruminococcaceae*. Community type B had the lowest proportion of *Bacteroides* and was dominated by genera within the *Firmicutes*. Compared to community type A, community type C had lower levels of *Bacteroides* but a higher abundance of *Alistipes*, *Faecalibacterium* and *Ruminococcaceae*, and also lacked *Prevotella*. The relative abundance profile of community type D showed lower levels of *Bacteroides* compared to community types A and C but a higher proportion of *Prevotella*. Another metagenomic analysis of intestinal microbiota samples from 96 healthy Russian adults living in urban and rural areas suggested the existence of additional microbial community structures within this cohort compared to large metagenomic studies in other countries [19]. These novel unique community structures identified in samples from Eastern Russia and rural regions were dominated by *Firmicutes* and *Actinobacteria* and for some samples the most abundant genera were unusual, for example *Bifidobacterium*, *Megamonas* (a genus within the family *Acidaminococcaceae* [20]), *Phascolarctobacterium*, *Lactobacillus* or *Akkermansia*. Host diet, cultural habits and socioeconomic status were suggested to contribute to the observed differences in microbiota compositions. It was hypothesised that broader global metagenomic analysis of intestinal microbiota diversity in rural and remote areas will identify even more variation in community structures, thus representing the diversity of human microbiota compositions before food industrialisation.

In addition to differences in the microbiota composition between proximal and distal parts of the GIT, distinct microbial compositions exist in the lumen and the mucosa [8,21]. However, knowledge regarding the mucosa-associated microbiota remains limited, as invasive methods are required for sampling [22]. The analysis of microbial communities of different colonic mucosal sites (caecum, ascending colon, transverse colon, descending colon, sigmoid colon, and rectum) suggested a pattern of patchiness and heterogeneity in the distribution of mucosal bacteria along the course of the colon, which may indicate the existence of microanatomic niches [8].

Generally, the mucosa-associated microbiota comprises a higher abundance of *Firmicutes* compared to *Bacteroidetes* [23]. The intestinal mucosa represents a glycan-rich environment, which is formed by the cell surface glycocalyx and the extracellular secreted mucus. Bacterial species colonising this milieu are specialised through the production of mucin-degrading enzymes and mucin-binding extracellular proteins. Although several mucin-degrading bacteria are increased in number during inflammation, which suggests they contribute to the disease pathogenesis, they are also part of the mucosa-associated microbiota in healthy humans [24]. Species described as mucin-degrading specialists include prominent commensals such as *Bacteroides thetaiotaomicron* [25] and* Akkermansia muciniphila* [26], both of which have been associated with protective effects* in vitro* and* in vivo* [27,28,29,30].

A simplified model of the human intestinal microbiota has demonstrated the specialisation of *B. thetaiotaomicron* to utilise host-derived mucin glycans [31]. Germ-free mice were colonised with *B. thetaiotaomicron* and *Eubacterium rectale* and microbial-microbial and microbial-host interactions were characterised. *B. thetaiotaomicron* adapts to the presence of *E. rectale* by increasing the expression of various genes involved in the harvest of host-derived mucin glycans that *E. rectale* is unable to utilise as a substrate. Furthermore, the bacterial signals produced by *B. thetaiotaomicron* induce the production of intestinal mucosal glycans. Adaptations of *E. rectale* in response to *B. thetaiotaomicron* include decreased production of its glycan-degrading enzymes and increased expression of selected amino acid and sugar transporters, and generation of butyrate, which is used by the intestinal epithelium. These results demonstrate the niche specialisation of the two bacterial species as the reciprocal effects on their metabolism enables them to utilise different nutrients.

The composition of the human intestinal microbiota is shaped by the influence of various factors, e.g., host genetics [32]. In addition, it is recognised that diet modulates the microbial composition in the intestine [33]. For instance, the influence of diet on microbiota composition is exploited in the use of prebiotics, currently defined as “selectively fermented ingredients that allow specific changes, both in the composition and/or activity in the intestinal microbiota that confers benefits upon host wellbeing and health” [34]. For example, the prebiotic inulin increased the proportion of *Bifidobacterium adolescentis* and *Faecalibacterium prausnitzii* in the human intestinal microbiota [35].

## 3. The Role of Commensal Bacteria in Human Health

Human health depends largely on the symbiotic benefits of the host-microbe relationship. Firstly, the intestinal microbiota has a protective function through the prevention of the colonisation of the mucosa by pathogens. This effect is often referred to as colonisation resistance and is achieved through various mechanisms [36]. Adherent bacteria of the intestinal microbiota occupy binding sites on the epithelium and thereby prevent the attachment and invasion of pathogenic bacteria into epithelial cells [37,38]. In addition, commensal bacteria can inhibit pathogenic microorganisms directly through production of antimicrobial substances such as bacteriocins [39], or indirectly by competing for available nutrients [40].

The intestinal microbiota provides energy and nutrients to the host through the breakdown of nondigestible products in the large intestine [41]. Additionally, intestinal bacteria play an important metabolic role through synthesising vitamins K and B_12_, folic acid [41] and short-chain fatty acids (SCFAs) such as acetate, butyrate and propionate [42]. Butyrate, in particular, has a central function as the main energy source for colonocytes. Moreover, butyrate exerts anti-inflammatory properties. For example, butyrate decreased pro-inflammatory cytokine expression in colonic biopsy specimens and lamina propria mononuclear cells isolated from patients with Crohn’s disease [43].

In addition, bacterial colonisation of the GIT is necessary for the normal postnatal development of the GIT. This has been demonstrated in experiments using germ-free animals that are raised in isolators to prevent colonisation of the GIT and other body surfaces by environmental microorganisms [44,45]. Studies have shown that the development of the GIT is impaired in these animals [46,47,48,49]. The advantage of the germ-free model is the ability to study the effect of colonisation with a single defined bacterial species or a complex microbiota, on physiological processes in the host [45]. The importance of bacterial colonisation is especially highlighted in the development of the immune system of the GIT, a process which is highly dependent on microbial colonisation, as discussed in more detail below [44]. Furthermore, the indigenous microbiota regulates intestinal angiogenesis, with adult germ-free mice displaying arrested capillary network formation [50]. The development of the vascular network could be initiated through inoculation with a complete microbiota harvested from conventional mice, but also by colonisation with one of the major members of the intestinal microbiota, *B. thetaiotaomicron* [50]. Recent findings have also demonstrated that commensal *Lactobacillus* spp. promote the proliferation of IECs, highlighting the importance of microbial stimuli [51]. This process is the result of the stimulation of NADPH oxidase 1-dependent reactive oxygen species generation by the bacteria, which in turn increases cellular proliferation [51].

## 4. Microbial Regulation of Intestinal Immune Homeostasis

Various regulatory adaptations exist to maintain the symbiotic relationship between humans and their intestinal microbiota [52]. In a healthy intestine, these adaptations prevent a constant activation of the immune system by commensal bacteria and food antigens but at the same time enable defence against pathogens. Commensal bacteria are critical for the development and function of the immune system and contribute to maintaining homeostasis in the GIT.

Immune cell populations and their respective functions vary greatly between the upper and the lower part of the GIT, as recently reviewed [53]. Immune responses within the small intestine focus on the defence against pathogens and tolerance towards food antigens. With the vast increase of the microbial density towards the distal locations of the GIT, the focus of the immune responses within the colon is the prevention of inflammatory responses against the commensal microbiota. First, the thicker mucus layer in the colon compared to the small intestine provides a physical barrier to prevent the entry of bacteria into the epithelium. In addition, the large numbers of mucosal immunoglobulin A producing plasma cells, interleukin-10 (IL-10) producing macrophages and regulatory T cells in the colon demonstrate the importance of regulatory immune functions within this part of the GIT [53].

### 4.1. Development and Function of the Immune System Depends on Microbial Colonisation

The importance of the intestinal microbiota in the development and function of the immune system is widely recognised [54]. Studies using germ-free animals have provided a valuable tool to investigate how commensal bacteria shape immune function. The absence of microorganisms in germ-free animals has been associated with an impaired development of the immune system compared to conventionally housed animals. For example, germ-free mice have poorly developed Peyer’s patches, lymphoid aggregates of the gut-associated lymphoid tissue [44]. Furthermore, germ-free mice have a reduced number of CD4^+^ T cells and immunoglobulin A producing B cells in the lamina propria compared to colonised animals [55,56]. Differences between germ-free and conventionally housed animals are not limited to the intestinal immune system. Germ-free animals also display an impaired development of systemic lymphoid structures with a reduction in B and T cells in the spleen and peripheral lymph nodes [44,57]. Numerous effects on non-immune systems of the GIT have also been reported. These include changes in morphology, absorptive function, electrolyte handling, bile metabolism, motility, and enteroendocrine and exocrine function as previously reviewed [45]. It has been shown that germ-free animals exposed to commensal bacteria develop an immune system similar to conventional animals within several weeks of inoculation [44].

In addition to the central role of the intestinal microbiota in the development of the immune system, an association between alterations in the composition of the microbiota, so-called dysbiosis, and inflammatory diseases has been reported [1,2,3,6]. For example, inflammatory bowel diseases are associated with a reduction in bacterial diversity, an increase in the abundance of *Bacteroidetes* and a reduction in the abundance of *Firmicutes* [58]. However, conflicting findings have also shown a decrease in the abundance of *Bacteroidetes* and *Firmicutes* in patients with inflammatory bowel diseases compared to healthy controls [59]. One explanation for these inconsistent findings may be differences in the samples analysed. For example, results obtained from compositional analysis of faecal samples were compared to that of mucosal tissue samples, which are known to harbour distinct microbial compositions [58,59]. In addition to the described phylum level changes, specific bacterial species were decreased in the intestinal microbiota of patients with inflammatory diseases, e.g., *F. prausnitzii* was reduced in inflammatory bowel diseases, irritable bowel syndrome and coeliac disease [60]. However, it remains unclear if intestinal dysbiosis is the cause or the consequence of disease pathogenesis [61].

Induction of inflammation by changes in the composition of the intestinal microbiota has been demonstrated using a colitis mouse model. Mice deficient in T-bet, a transcription factor involved in the regulation of mucosal immune responses to commensal bacteria, developed spontaneous ulcerative colitis in the absence of adaptive immunity [62]. Remarkably, this colitis was both vertically and horizontally transmissible to genetically intact hosts as shown in cross-fostering and co-housing experiments, respectively. The authors suggested that the loss of T-bet supports the development of a colitogenic microbial community in this colitis mouse model. The finding that colitis was communicable to wild-type mice highlights the significance of commensal bacteria in controlling immune functions.

### 4.2. The Role of Innate Signalling in Maintaining Immune Homeostasis

Bacteria in the intestinal lumen are recognised by IECs and immune cells via the expression of pattern recognition receptors (PRRs). These receptors recognise common structures on microbial surfaces, so-called pathogen-associated molecular patterns (PAMPs), a term which is increasingly replaced by microbe-associated molecular patterns (MAMPs), as these structures are also part of non-pathogenic and commensal microbes [13]. PRRs comprise several families of receptors, namely the nucleotide oligomerisation domain-like receptors, retinoic acid inducible gene I-like receptors and Toll-like receptors (TLRs). The best characterised PRRs are the TLRs and nucleotide oligomerisation domain-like receptors [63]. Some of the 13 TLRs reported to be found in mice and humans to date are localised in the cell membrane, others in endosomal membranes, in order to recognise extracellular and endocytosed MAMP, respectively [63]. TLR2, 4, 5 and 9 recognise common bacterial and fungal structures, whereas TLR3, 7 and 8 are primarily involved in viral detection. TLR stimulation triggers downstream signalling cascades leading to the activation of transcription factors, which induce the release of pro-inflammatory cytokines and chemokines resulting in the induction of inflammatory immune responses against pathogens [13].

TLR signalling is critical in the innate defence against pathogens. Constant activation of the immune system of the GIT through the recognition of the resident microbiota would be pathological. Therefore, the host needs to be able to distinguish between commensals and pathogens, and balance tolerance and immunity. This is partly achieved through the regulated expression and distribution of PRRs in the GIT [13,63]. For example, IECs express low levels of TLR2 and TLR4 under healthy conditions, reflecting one of the mechanisms for the immunological hyporesponsiveness to commensal bacteria [64,65]. Differences in the expression of PRRs between apical and basolateral surfaces of IECs are another way to distinguish between pathogenic and commensal bacteria. TLR5, which recognises bacterial flagellin, is only expressed on the basolateral side of IECs. It has been shown that pathogenic *Salmonella*, but not commensal *E. coli*, translocate flagellin across the epithelia. Thus, only pathogenic *Salmonella* activates TLR5 and induces epithelial pro-inflammatory gene expression [66].

Despite these mechanisms to reduce TLR signalling in the healthy intestine, it is becoming increasingly evident that a basal level of TLR signalling induced by luminal microbiota contributes to homeostasis. Evidence for this homeostatic influence of TLR signalling on the intestinal epithelium mainly originated from work in TLR knockout mice. For instance, mouse models with the genes for TLR2, 4, 9 or the adaptor protein MyD88 knocked out have increased susceptibility to colitis induced by dextran sodium sulphate (DSS) [67,68].

One example that demonstrates the contribution of TLR signalling to maintain immune homeostasis is the signalling upon TLR2 activation. This receptor activation can result in pro- and anti-inflammatory responses, which is dependent upon the interaction of TLR2 with multiple co-receptors [69]. TLR2 recognises surface structures of Gram-negative and Gram-positive bacteria and yeast, such as lipoproteins, lipoteichoic acid and zymosan, respectively. TLR2 occurs in the form of heterodimers with different co-receptors; TLR1 and TLR6. It has been reported both* in vitro* and* in vivo*, that stimulation of the TLR2/6 heterodimer results in the differentiation of dendritic cells (DCs) towards a tolerogenic phenotype and an increase of IL-10 producing regulatory T cells [70]. However, the activation of the TLR2/1 heterodimer results in increased production of IL-12p40 and less IL-10 by DCs and promotes the differentiation of inflammatory T cells. The pro- and anti-inflammatory responses upon activation of TLR2/1 and TLR2/6, respectively, is a consequence of different cell signalling pathways activated by the different heterodimers. The pro-inflammatory response upon TLR2/1 activation is induced by p38-MAPK activation. In contrast, the regulatory response upon TLR2/6 activation is dependent on c-Jun *N*-terminal kinase activation [70].

In the case of the example above, the differentiation of tolerogenic DCs that promote regulatory IL-10 immune responses was induced through TLR2/6 activation by a virulence factor of the pathogen *Yersinia pestis* [70]. This mechanism allows the pathogen to escape immune defence mechanisms through blocking inflammation. This might also be a mechanism used by commensal bacteria to contribute to intestinal immune homeostasis.

### 4.3. Microbial Modulation of the Function of Intestinal Epithelial Cells and Immune Cells

Recent studies have demonstrated a more complex role for IECs than simply forming a physical barrier between the lumen and underlying tissues. IECs also maintain immune homeostasis through sensing the microbial environment via the expression of PRRs and subsequently regulating the function of antigen-presenting cells and lymphocytes [12,63]. IEC function is influenced by commensal bacteria which are able to actively modulate signalling in IECs [12]. These modulations provide a mechanism for commensal bacteria to be recognised by IECs without activating a pro-inflammatory immune response, and therefore allowing the co-existence of humans and their intestinal microbiota [13].

Some commensal bacteria are able to inhibit PRR-mediated NF-κB activation [27,71]. Under steady-state conditions, this transcription factor is bound by its inhibitor IκB, which prevents its nuclear translocation. The classical NF-κB activation following receptor stimulation is obtained through tightly regulated phosphorylation, ubiquitination and proteasomal degradation of IκB, resulting in the translocation of NF-κB into the nucleus where it activates the transcription of inflammatory cytokines and chemokines [72]. Experiments with nonvirulent *Salmonella* strains have shown that these commensal bacteria inhibit this pathway by blocking IκB ubiquitination and subsequent degradation, therefore preventing NF-κB activation and maintaining epithelial hyporesponsiveness towards the luminal microbiota [71].

Commensal-derived metabolites are also known to modulate innate immune responses providing another mechanism for maintaining intestinal immune homeostasis and tolerance. Butyrate, a short-chain fatty acid and one of the main end products of fermentation of dietary fibre by intestinal bacteria, is a major source of energy for IECs. Moreover, butyrate can exert direct immunomodulatory and anti-inflammatory effects [73]. For instance, butyrate inhibits histone deacetylase activity and thereby suppresses proteasome activity by reducing the expression levels of selected proteasome subunits. This results in the inhibition of the proteasomal degradation of IκB and consequently limits NF-κB activation [74].

Furthermore, IEC recognition of commensal bacteria triggers the production of several immunoregulatory molecules, for example thymic stromal lymphopoietin (TSLP) and transforming growth factor β (TGFβ) [75,76]. These signals, in turn, promote the development of mucosal immune cells with tolerogenic properties. This cross-talk between commensal bacteria, IECs and immune cells is important for maintaining homeostasis and limiting uncontrolled inflammation in the GIT [77].

## 5. Commensal Obligate Anaerobes Regulate Immune Homeostasis

The central role of microbial colonisation in maintaining immune homeostasis is well known [52]. However, knowledge regarding the contributions made by obligate anaerobic bacterial species is still limited. Numerous studies have demonstrated the immunomodulatory effects of commensal and probiotic lactobacilli, as previously reviewed [78,79]. Lactobacilli are common members of the infant intestinal microbiota, whereas their abundance in adults is low, with most *Lactobacillus* spp. colonising the upper GIT [80]. However, the colon, that part of the GIT with the highest bacterial density, is predominated by obligate anaerobic species [8]. The health promoting effect of lactic acid bacteria, including lactobacilli, was discovered more than a century ago by Elie Metchnikoff [81]; a finding that established the origin of the probiotic concept. Due to the economic success of probiotics, research on lactobacilli has greatly accelerated over the last few decades to the detriment of study on other bacteria. Technical difficulties have also been a limiting factor in the investigation of host-microbe interactions. Oxygen-tolerant bacteria such as lactobacilli can be readily co-cultured with human cells to enable study of the mechanism of action behind the effects of these bacteria. However, obligate anaerobic bacteria cannot survive in the presence of oxygen and it is therefore impossible to co-culture these bacteria with oxygen-requiring intestinal cells in conventional systems. Consequently, little is known about the exact mechanisms behind the effects of commensal obligate anaerobes, which means that the effects of the majority of the intestinal microbiota remain unknown.

**Figure 1:**
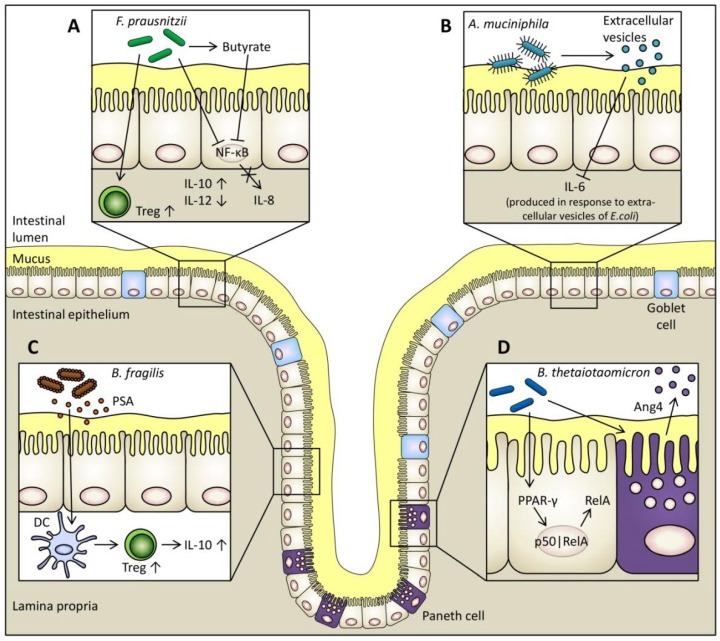
Proposed immunomodulatory mechanisms of four examples of obligate anaerobic commensals. (**A**) The supernatant of *F. prausnitzii* blocks the activation of transcription factor NF-κB which inhibits the production of pro-inflammatory IL-8 by intestinal epithelial cells (IECs) [83]. *F. prausnitzii* is one of the major butyrate producers in the large intestine. Butyrate also blocks NF-κB activation [74]. Furthermore, *F. prausnitzii* increases the differentiation of regulatory T cells (Treg) [84] and the production of anti-inflammatory IL-10 by peripheral blood mononuclear cells and decreases the production of pro-inflammatory IL-12 [83,84]. (**B**) Extracellular vesicles produced by *A. muciniphila* block the production of pro-inflammatory IL-6 from colon epithelial cells in response to extracellular vesicles of *Escherichia coli* [29]. (**C**) A surface molecule of *B. fragilis*, polysaccharide A (PSA), exerts immunomodulatory functions within the GIT. PSA is recognised by DCs which induces increased production of regulatory T cells and anti-inflammatory IL-10 [85,86]. (**D**) *B. thetaiotaomicron* causes the PPAR-γ dependent nuclear export of the NF-κB transcriptionally active subunit RelA in IECs which attenuates pro-inflammatory cytokine expression [27]. *B. thetaiotaomicron* also stimulates the release of the antimicrobial peptide Ang4 by Paneth cells thereby maintaining mucosal barrier function [28].

The use of high-throughput and next-generation sequencing techniques has rapidly increased our knowledge of the intestinal microbiota and its significance in human health and disease [82]. In addition, various studies have shown that dysbiosis is associated with diseases involving chronic intestinal inflammation (e.g., inflammatory bowel diseases) and that a decrease in the abundance of certain bacterial species occur in these diseases [1,2,3,6]. It has therefore been hypothesised based on results of* in vitro* and* in vivo* studies that these species have a positive effect on human health. Taken together, this information could lead to a broadening of the classical probiotic concept, where currently most of the bacterial strains belong to the lactic acid bacteria, to include a selection of strains from a potential next generation of probiotics originating from indigenous bacteria [82]. Examples of obligate anaerobic commensal bacteria that may exert beneficial effects on human health, as indicated by the results of* in vitro* and animal studies, are described below. The known immunoregulatory effects of these species are summarised in Figure 1.

### 5.1. Faecalibacterium prausnitzii

The obligate anaerobe *F. prausnitzii*, a major member of the clostridial cluster IV within the phylum *Firmicutes*, is regarded as one of the most prevalent bacteria within the human GIT [83,87] and accounts for 1.4% to 5.9% of the total faecal microbiota of healthy adults [88]. Originally isolated in 1922 by C. Prausnitz and named *Bacillus mucosus anaerobius*, the bacterium has been reclassified several times. After being named *Fusobacterium prausnitzii* in 1973 [89], the bacterium was further reclassified to its current name *Faecalibacterium prausnitzii* in 2002 [87]. The complete genome sequences of two strains, *F. prausnitzii* L2-6 (PRJNA197183, PRJNA45961) and *F. prausnitzii* SL3/3 (PRJNA197157, PRJNA39151), are available. *F. prausnitzii* is a Gram-negative bacterium, even though phylogenetic analysis based on 16S rRNA sequencing indicates that *F. prausnitzii* is related to the Gram-positive bacteria of the clostridial cluster IV [87]. Furthermore, *F. prausnitzii* is non-spore forming, non-motile and rod-shaped [87]. This commensal bacterium produces butyrate, d-lactate and formate as fermentation products and utilises acetate.

Recent studies have shown a reduced abundance of *F. prausnitzii* in patients with inflammatory bowel diseases compared to healthy controls [3,90,91,92,93]. Furthermore, a reduction of this bacterium is associated with a higher risk of postoperative recurrence of ileal Crohn’s disease [83]. *F. prausnitzii* is also reduced in patients with irritable bowel syndrome [94], colorectal cancer [95] and coeliac disease [6]. Based on these findings, it has been hypothesised that the presence of *F. prausnitzii* acts as a protective factor for the intestinal mucosa and promotes intestinal health. Nevertheless, the mechanisms of the effects of *F. prausnitzii* are only partially understood and require further exploration. *F. prausnitzii* has also been suggested as a promising candidate for treating inflammatory bowel diseases [83,84,96]. This is supported by the finding that administering *F. prausnitzii* protects against experimental colitis in animal models [83,84], but has not yet been tested in human clinical studies.

Only one study has challenged the model of a protective role for *F. prausnitzii*. In contrast to adult Crohn’s disease, where a reduced abundance of *F. prausnitzii* was observed, this species was increased in the colonic mucosa of children with newly diagnosed Crohn’s disease [97]. The authors suggested a more dynamic function of *F. prausnitzii* than described in previous studies. However, it could be possible that paediatric inflammatory bowel diseases are associated with different changes in microbial composition compared to adult disease.

*F. prausnitzii* may contribute to the maintenance of intestinal health through several mechanisms. It may exhibit its beneficial effects indirectly by providing colonocytes with their most important energy source, butyrate, and therefore strengthen the epithelium [87]. However, direct anti-inflammatory effects of *F. prausnitzii* have also been demonstrated both* in vitro* and* in vivo*. In a human* in vitro* cell model, secreted metabolites from *F. prausnitzii* A2-165 (DSM 17677) were able to block NF-κB activation and IL-8 production [83], which was not due to bacterial butyrate production. Additionally, the stimulation of peripheral blood monocytes (PBMCs) by *F. prausnitzii* has been shown to increase production of anti-inflammatory IL-10 and decrease production of pro-inflammatory IL-12. The same group demonstrated a reduction in 2,4,6-trinitrobenzenesulfonic acid (TNBS) induced colitis activity in mice orally administered *F. prausnitzii* or its culture supernatant [83].

Recently, a similar study provided new insights into the anti-inflammatory and immunomodulatory properties of *F. prausnitzii* [84]. Both *F. prausnitzii* (ATCC 27766) and its culture supernatant induced anti-inflammatory cytokine production in human PBMCs and a rat model of TNBS-induced colitis. Cytokine concentrations were quantified in PBMC culture supernatants and rat blood serum. The *F. prausnitzii* supernatant exhibited the strongest anti-inflammatory effect and showed improved protection against experimental colitis compared to the other treatment groups, which also included a probiotic *Bifidobacterium longum* (strain provided by Shanghai Sine Pharmaceutical Company, China). Furthermore, *F. prausnitzii* and its culture supernatant increased the production of regulatory T cells [84].

In mice with DSS-induced colitis, the *F. prausnitzii* culture supernatant improved intestinal barrier integrity by affecting paracellular permeability [98], as measured by ^51^chromium (Cr)-EDTA transport. The paracellular passage of ^51^Cr-EDTA was increased in the ileal mucosa of mice treated with DSS compared to control animals. Treatment with the *F. prausnitzii* culture supernatant resulted in decreased passage of ^51^Cr-EDTA, indicating improved intestinal barrier integrity. This effect was suggested to contribute to the reduction of the severity of DSS-induced colitis in mice [98].

A recent study has investigated the effect of viable and dead *F. prausnitzii* A2-165 (DSM 17677) on global gene expression of Caco-2 cells [99]. Viable *F. prausnitzii* exerted differential effects on Caco-2 cells compared to dead bacteria with most of the differences observed in genes involved in immune pathways. Functional analysis using Ingenuity^®^ Pathway Analysis, a bioinformatic tool used to analyse and interpret the biological meaning of gene expression data, predicted a decrease in functions belonging to the “inflammatory response” and “immune cell trafficking” categories in Caco-2 cells treated with live *F. prausnitzii* compared to untreated Caco-2 cells. These results suggest the production of an anti-inflammatory compound by live *F. prausnitzii* [99].

Taken together, the results of the above-described studies indicate a role of *F. prausnitzii* in maintaining intestinal homeostasis. The decreased abundance of *F. prausnitzii* in diseases including inflammatory bowel diseases and coeliac disease may contribute to the reduction in commensal bacteria-mediated, anti-inflammatory activities in the intestinal mucosa [93].

### 5.2. Bacteroides thetaiotaomicron

*B. thetaiotaomicron*, a Gram-negative obligate anaerobic organism belonging to the phylum *Bacteroidetes*, is a prevalent member of the human intestinal microbiota [100]. A study investigating the diversity of the human intestinal microbiota showed that within this phylum, which comprises 48% of all bacterial 16S rDNA sequences analysed, *B. thetaiotaomicron* accounted for 13% [8]. The complete genome sequence of one strain, *B. thetaiotaomicron* VPI-5482 (PRJNA62913), is available [101]. Another strain, *B. thetaiotaomicron* dnLKV9 (PRJNA207381), is currently available as a scaffold. This revealed that *B. thetaiotaomicron* may be able to utilise a large variety of complex polysaccharides as substrates [100,101]. Furthermore, the organism is able to adapt to the changing colonic environment through regulation of the expression of genes involved in its polysaccharide utilisation machinery [31]. These characteristics confer an advantage for survival in the changing host intestinal lumen and may explain the high abundance of *B. thetaiotaomicron* in the human intestinal microbiota [100,101]. The prevalence of *B. thetaiotaomicron* is reduced in adult and pediatric patients with inflammatory bowel diseases [1,102,103], indicating a role for this commensal bacterium in maintaining intestinal health. However, compared to *F. prausnitzii*, evidence for this association is weak with only a few studies reporting this observation. The reduced abundance of *B. thetaiotaomicron* observed in patients with inflammatory bowel diseases could also indicate an increased susceptibility of this bacterium to inflammatory conditions.

A number of studies indicate that, in addition to providing nutrients to the host through the digestion of complex carbohydrates, *B. thetaiotaomicron* also influences the host directly via interaction with IECs. For example, the colonisation of germ-free mice with *B. thetaiotaomicron* VPI-5482 (ATCC 29148) increased the expression of several host genes important for intestinal functions including nutrient absorption, mucosal barrier function, production of angiogenic factors and motility [104].

Additionally, *B. thetaiotaomicron* influences host immune responses. For example, *B. thetaiotaomicron* attenuates pro-inflammatory cytokine expression by inducing the expression of peroxisome proliferator-activated receptor-γ (PPAR-γ) in Caco-2 cells, which promotes nuclear export of the NF-κB transcriptionally active subunit; v-rel avian reticuloendotheliosis viral oncogene homolog A (RelA) [27]. Furthermore, *B. thetaiotaomicron* stimulates the production of antimicrobial peptides or proteins by Paneth cells [28]. Antimicrobial peptide angiogenin 4 (Ang4) released by Paneth cells in germ-free mice can be induced by colonisation with *B. thetaiotaomicron* VPI-5482 (ATCC 29148)*.* This antimicrobial protein kills Gram-positive pathogens such as *Listeria monocytogenes*; however, the Gram-negative commensal *B. thetaiotaomicron* is less sensitive with only a 30% reduction of viable cells upon exposure to Ang4. Stimulation of the production of antimicrobial proteins, e.g., Ang4, represents a mechanism of how commensal bacteria such as *B. thetaiotaomicron* can contribute to maintaining mucosal barrier function [28].

There is preliminary evidence to suggest that *B. thetaiotaomicron* may be effective in the treatment of infectious diarrhoea. For example, *B. thetaiotaomicron* VPI-5482 (ATCC 29148) contributed to the prevention of rotavirus infection* in vitro* [105]. However, clinical evidence for this potentially protective effect of *B. thetaiotaomicron* on rotavirus infection is not yet available.

In addition to the effects of a single species, the interplay between two major commensal bacteria, *F. prausnitzii* and *B. thetaiotaomicron*, promotes colonic mucosal homeostasis [106]. Germ-free rats mono-associated with *B. thetaiotaomicron* VPI-5482 (ATCC 29148) had increased goblet cell differentiation and expression of mucus-related genes. This stimulation of the secretory lineage and increase in mucus production was reduced in germ-free rats di-associated with *B. thetaiotaomicron* and *F. prausnitzii* A2-165 (DSM 17677). The authors suggested that the balance between these species could contribute to colonic mucosal homeostasis [106].

### 5.3. Bacteroides fragilis

*B. fragilis* is an obligate anaerobic bacterium within the phylum *Bacteroidetes* and a common member of the human intestinal microbiota [107,108]. Compared to the other members of the *Bacteroidetes*, *B. fragilis* is the least abundant species in the faecal microbiota, accounting for only 0.6% of the bacteria present in stool [108]. It is generally a commensal bacterium, however when translocated into the bloodstream or extra-intestinal tissue after surgery, disease or trauma it can cause serious infections [109].

*B. fragilis* exerts immunomodulatory functions within the GIT. Mice with experimental colitis induced by colonisation with the pathobiont *Helicobacter hepaticus* were protected from disease when co-colonised with *B. fragilis* [85]. This protection was shown to depend on a surface molecule of *B. fragilis*, polysaccharide A (PSA), as the purified molecule alone prevented intestinal inflammation* in vivo.* PSA induced the production of anti-inflammatory IL-10* in vitro* which was suggested to be required for the inhibition of inflammation [85]. PSA is packaged into outer membrane vesicles (OMVs) when released by the bacterium [86]. These OMVs containing PSA protected mice with TNBS-induced colitis. The OMV-associated PSA is recognised by DCs through TLR2 which induces increased production of regulatory T cells and anti-inflammatory cytokines [86]. Results of a recent study revealed that a specific DC subset namely plasmacytoid DCs (PDCs) are the mediators behind the PSA-induced protection against experimental colitis in mice [110]. This protection was dependent on both innate and adaptive immune mechanisms and TLR2-mediated. The exposure of PDCs with PSA increased the production of anti-inflammatory IL-10 by CD4^+^ T cells.

Interestingly, PSA released by *B. fragilis* also conferred protection from inflammation in extra-intestinal tissue [111]. This immunomodulatory molecule protected against central nervous system demyelination and inflammation during experimental autoimmune encephalomyelitis, which is used as an animal model for multiple sclerosis. The protective effect was mediated through TLR2 signalling. These results indicate that the intestinal microbiota may influence the susceptibility of the host towards extra-intestinal autoimmune disorders.

### 5.4. Akkermansia muciniphila

The obligate anaerobe* A. muciniphila* belongs to the phylum *Verrucomicrobia* and is part of the mucosa-associated microbiota because of its ability to produce mucus-degrading enzymes [26]. *A. muciniphila* is a common member of the human intestinal microbiota; its abundance in the colon ranges between 1% to 3% of the bacterial population [112].

Several studies have shown a reduced abundance of *A. muciniphila* in humans with various conditions or diseases. For example, *A. muciniphila* is less abundant in obese children [113] and its abundance decreases with increasing body weight gain in pregnant women [114]. *Akkermansia* spp. are also reduced in children with atopic diseases [115]. The reduction of *A. muciniphila* in patients with ulcerative colitis and Crohn’s disease compared to its high abundance in the mucosa-associated microbiota of healthy controls led to the suggestion that this species might play a protective or anti-inflammatory role in the intestinal mucosa [24]. Furthermore, *A. muciniphila* has been proposed as a marker for a healthy intestine due to its reduction in disease but high abundance in the healthy mucosa [116].

A potential protective role for *A. muciniphila* in inflammatory bowel diseases was investigated in a recent study. Extracellular vesicles derived from *A. muciniphila* conferred protective effects in mice with DSS-induced colitis such as amelioration of body weight loss, colon length (decreased in mice treated with DSS), and inflammatory cell infiltration of the colon wall [29]. Furthermore, pre-treatment of a colonic epithelial cell line (CT26) with *A. muciniphila*-derived extracellular vesicles resulted in an anti-inflammatory effect as shown by decreased production of the pro-inflammatory cytokine IL-6 induced by extracellular vesicles of a pathogenic strain of *Escherichia coli* [29].

The influence of *A. muciniphila* on metabolic functions of the host and the association of this bacterium with obesity has also been investigated in a recent* in vivo* study. *A. muciniphila* was reduced in genetically obese mice (ob/ob mice) and mice fed a high-fat diet [30]. Mice fed a high-fat diet supplemented with oligofructose, a prebiotic, had a normal abundance of *A. muciniphila* and an improvement in metabolic disorders, endotoxemia and adipose tissue inflammation. In addition, when mice fed a high-fat diet were given viable *A. muciniphila* Muc^T^ (ATTC BAA-835), this resulted in normalisation of the metabolic endotoxemia, fat-mass gain, insulin resistance and adipose tissue inflammation; all of which were symptoms induced by the high-fat diet [30]. Based on the results of this study, the authors suggested a potential application of *A. muciniphila* in the prevention and/or treatment of obesity and associated metabolic disorders [30].

Despite the increasing interest in *A. muciniphila* over the last few years, knowledge regarding the precise mechanisms of the host-microbial interaction causing these beneficial effects remains to be elucidated and will require further exploration. Furthermore, recent studies have mainly focused on the protective role of *A. muciniphila* in obesity and type-2 diabetes but only a few studies have thus far addressed the link between this species and inflammatory disorders such as inflammatory bowel diseases. In addition, clinical evidence for a protective function of *A. muciniphila* does not yet exist. Furthermore, it is not clear if *A. muciniphila* only exerts beneficial effects or if this bacterium may also be associated with potentially harmful effects under certain conditions. *A. muciniphila* releases sulphate during fermentation of mucin [26] which could be utilised either by this species or other sulphate-reducing bacteria to produce hydrogen sulphide. The increased availability of sulphate may lead to an increased abundance of sulphate-reducing bacteria which have been associated with inflammation in genetically susceptible hosts [117]. However, if *A. muciniphila* exerts any potential inflammatory effects these may be dependent on competition with other bacteria specialised in mucin-degradation [31], the composition of the intestinal microbial ecosystem [118] and/or host susceptibility [117].

### 5.5. Segmented Filamentous Bacteria

Segmented filamentous bacteria (SFB), first observed in the ilea of mice and rats [119], are obligate anaerobic bacteria that have been identified to populate various vertebrates including humans [120]. Metagenomic comparisons indicated that SFB are closely related to *Clostridium* spp. [121]. Thus far it is impossible to culture SFB using current standard culture techniques [122]. SFB have been shown to firmly attach to IECs by one end of the filament [119].

The ability to mono-colonise germ-free mice with SFB [123] set the starting point for numerous studies investigating the interactions between this commensal organism and its host. These studies have revealed a critical role for SFB in the maturation of the immune system. Mono-colonisation of germ-free mice with SFB increased the number of lymphoid and IgA-secreting cells in the intestinal mucosa and elevated IgA titres in serum and secretions in comparison to germ-free mice [124]. Furthermore, SFB influence the development and activation of T helper cells. In particular, they induce the development of intestinal Th17 cells [125]. This activation of a Th17 response conferred protective effects to mice through increased colonisation resistance to the intestinal pathogen *Citrobacter rodentium* [125]. SFB have also been shown to simultaneously induce Th1, Th2, Th17, and regulatory T cell responses in mono-colonised mice [126]. Remarkably, colonisation of germ-free mice with this single species induced a similar coordinated maturation of intestinal T cell responses as induced by the colonisation with the whole mouse microbiota. This effect was not observed when different bacteria were used, which points out the key role of SFB in shaping immune functions in the intestine.

## 6. Dual-Environment Co-Culture Models to Study Host-Microbe Interactions

**Figure 2:**
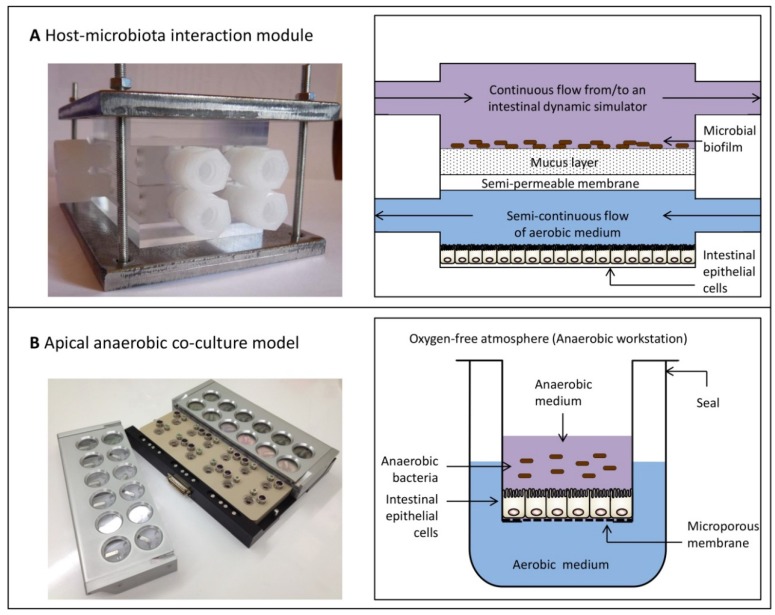
Comparison of two examples of dual-environment co-culture models. (**A**) The Host-Microbiota Interaction (HMI) module enables the co-culture of a complex microbial community, including obligate anaerobic bacteria, with enterocyte-like cells [129,130]. This* in vitro* model consists of two compartments, the upper compartment representing the luminal side containing a complex microbial community and the lower compartment containing enterocyte-like cell lines. These two compartments are separated by a polyamide semipermeable membrane and a mucus layer. The HMI module can be used to investigate host-microbiota interactions for up to 48 h. (**B**) The apical anaerobic co-culture model also contains two compartments. The upper compartment contains anaerobic medium to enable the survival of obligate anaerobic bacteria and the lower compartment is filled with aerobic medium to enable the survival of oxygen-requiring human IECs. In this model the two compartments are separated by a microporous membrane, on which IECs are seeded. By diffusion through the microporous membrane, IECs receive oxygen from the lower aerobic compartment. The apical anaerobic co-culture model has been validated to study host-microbial interactions for up to 12 h [99]. Photograph of the HMI module is used with permission from Marzorati* et al.* [130], Agro Food Industry Hi-Tech, Tekno Scienze © 2012. Schematic of the HMI module is adapted from Marzorati* et al.* [129], BMC Microbiology, published by BioMed Central, 2014.

Knowledge concerning the exact mechanism(s) behind the effects of intestinal bacteria on the human epithelia remains limited due to the technical difficulties of co-culturing obligate anaerobic bacteria with oxygen-requiring human cells. Using conventional co-culture systems, it is only possible to test the effects of non-viable obligate anaerobes in co-culture with IEC lines. Although some biological effects can be elicited by non-viable bacteria through interactions with surface structures, other effects require live bacteria to generate responses which are indicated to be caused by secreted bacterial metabolites [127]. For example, a study investigating the effect of oxygen-tolerant *Lactobacillus reuteri* on the human IEC lines T84 and HT29 showed these bacteria had an anti-inflammatory effect, as demonstrated by the inhibition of TNF-α induced IL-8 expression [128]. This effect was only observed with live *L. reuteri*; neither heat-killed nor γ-irradiated bacteria induced a response in IECs. To test the effect of live and metabolically active obligate anaerobic bacteria, including the species described in this review,* in vitro* systems that enable the co-culture of live obligate anaerobes with oxygen-requiring human cells are needed. Several models have recently been established that allow this dual-environment co-culture with the aim of studying the mechanisms of action of obligate anaerobic bacteria. A comparison of two examples of recently developed models is shown in Figure 2. These models can be used to study the effect of obligate anaerobes on host immune responses.

Recently, Marzorati* et al.* [129] developed the Host-Microbiota Interaction (HMI) module, a device that enables the co-culture of a complex microbial community, including obligate anaerobic bacteria, with enterocyte-like cells (Figure 2A). The HMI module can be used in combination with intestinal dynamic simulation, where digestion processes in the stomach, small intestine and colon are simulated in several reactors. Furthermore, the last compartment of this dynamic* in vitro* simulator, which mimics the ascending colon, is inoculated with faecal microbiota. In combination with this continuous culture system, the HMI module can be utilised to study bacterial adhesion to the mucus layer under relevant shear forces. It also allows the exchange of signals and metabolites between the two compartments, and through the diffusion of oxygen from the lower compartment, it creates microaerophilic conditions on the bottom of the microbial biofilm. Consequently, it is able to mimic the physiological conditions of the GIT. Moreover, due to the separation of the two compartments by the mucus layer, this model is more representative of the GIT compared to co-culture systems that allow direct contact between bacteria and host cells.

Direct contact between live obligate anaerobic bacteria and human cell lines occurs in a novel dual-environment co-culture model which has been developed and validated in our laboratory [99]. This* in vitro* model, referred to as the apical anaerobic co-culture model, utilises a co-culture chamber, which is used inside an anaerobic workstation that has an oxygen-free atmosphere (Figure 2B). The apical anaerobic co-culture model can be used to determine the effect of bacteria on intestinal barrier function as the co-culture chamber contains built-in electrodes to monitor trans-epithelial electrical resistance across cell monolayers, a well-established measurement for barrier integrity [131]. Furthermore, each well of the co-culture chamber is equipped with septa to allow sampling of the basal media without altering the environment. Using paracellular tracers, e.g., [^3^H]-mannitol, the effect of anaerobic bacteria on the small molecule paracellular permeability of cell monolayers can also be determined, which represent another indicator of tight junction integrity [132]. Mucus-secreting cell lines such as HT29-MTX [133] could also be included in this model in order to separate bacteria from human IECs by a mucus layer, which is more representative of the GIT. The apical anaerobic co-culture model also allows the effects of individual obligate anaerobes on intestinal immune homeostasis to be determined, where methods are currently under development. For example, this will allow the maturation and phenotype of immature DCs upon exposure to obligate anaerobes to be ascertained. In addition, this novel model could be used to study the complex interactions between IECs, immune cells and obligate anaerobic bacteria through the co-culture of these three players in a spatial distribution similar to the situation* in vivo*. This adaption to our model system is also currently under development.

In addition to the two examples of dual-environment co-culture models described above, various recently developed* in vitro* models of the intestine exist that could be adapted to be used under dual-environment conditions. The “human gut-on-a-chip” consists of two microfluidic channels that are separated by a porous flexible membrane coated with extracellular matrix on which Caco-2 cells are seeded [134]. In order to mimic the physiological conditions in the intestine, the Caco-2 cells are exposed to low shear stress induced by fluid flow and peristalsis-like motions. This gut-on-a-chip was used for the co-culture of Caco-2 cells with the oxygen-tolerant commensal bacterium *Lactobacillus rhamnosus* GG for over one week on the apical surface without negative effects on the viability of the cells [134]. This model could be modified to include the dual-environment conditions to be able to study the effects of obligate anaerobic bacteria on intestinal functions.

Another recently developed* in vitro* model that closely mimics the physiology of the intestine are the three-dimensional “mini-intestines” derived from isolated human intestinal epithelial stem cells from the small intestine or colon [135,136]. The so-called enteroids contain all four types of normal epithelial cells. Currently, intestinal enteroids are grown in three-dimensional cultures to be used in functional studies; however, murine enteroids were also successfully grown as polarised monolayers [137]. Moreover, preliminary tests were performed with human enteroids seeded on porous Transwell membranes coated with a fibronectin-based peptide [138]. This resulted in the formation of confluent polarised monolayers with a high trans-epithelial electrical resistance (600–1000 Ω cm^2^). Intestinal enteroids grown on Transwell inserts could be used in combination with the technology of the apical anaerobic co-culture model (Figure 2B) to co-culture these “mini-intestines” with obligate anaerobic bacteria. Compared to Caco-2 cells, which are currently used in the apical anaerobic co-culture model, intestinal enteroids would be more representative for the physiological conditions in the intestine.

## 7. Conclusions

It is clear that commensal bacteria play an important role in maintaining intestinal immune homeostasis. However, information regarding the mechanisms of action behind these effects is limited, partially due to technical difficulties in co-culturing obligate anaerobes with host cells when using conventional models. Using the novel co-culture systems we have described here, it will now be possible to gain insight into these complex host-microbiota interactions and to understand the mechanisms behind the beneficial contribution of commensal bacteria to human health. The next steps will be confirming these* in vitro* effects using animal studies and, ultimately testing these bacteria in robust human clinical studies. This knowledge may lead to new approaches for improving human health and reducing the risk of diseases associated with a disturbed microbiota composition and inflammation.
